# Adolescents’ neural reactivity to parental criticism is associated with diminished happiness during daily interpersonal situations

**DOI:** 10.1093/scan/nsad020

**Published:** 2023-04-01

**Authors:** Kiera M James, Stefanie L Sequeira, Ronald E Dahl, Erika E Forbes, Neal D Ryan, Jill Hooley, Cecile D Ladouceur, Jennifer S Silk

**Affiliations:** Department of Psychology, University of Pittsburgh, Pittsburgh, PA 15260, USA; Department of Psychology, University of Pittsburgh, Pittsburgh, PA 15260, USA; School of Public Health, University of California - Berkeley, Berkeley, CA 94720, USA; Department of Psychiatry, University of Pittsburgh, Pittsburgh, PA 15260, USA; Department of Psychiatry, University of Pittsburgh, Pittsburgh, PA 15260, USA; Department of Psychology, Harvard University, Cambridge, MA 02138, USA; Department of Psychiatry, University of Pittsburgh, Pittsburgh, PA 15260, USA; Department of Psychology, University of Pittsburgh, Pittsburgh, PA 15260, USA

**Keywords:** neuroimaging, affective salience network, parental criticism, ecological momentary assessment, emotion

## Abstract

The goal of this study was to examine the relation between real-world socio-emotional measures and neural activation to parental criticism, a salient form of social threat for adolescents. This work could help us understand why heightened neural reactivity to social threat consistently emerges as a risk factor for internalizing psychopathology in youth. We predicted that youth with higher reactivity to parental criticism (*vs* neutral comments) in the subgenual anterior cingulate cortex (sgACC), amygdala and anterior insula would experience (i) less happiness in daily positive interpersonal situations and (ii) more sadness and anger in daily negative interpersonal situations. Participants (44 youth aged 11–16 years with a history of anxiety) completed a 10-day ecological momentary assessment protocol and a neuroimaging task in which they listened to audio clips of their parents’ criticism and neutral comments. Mixed-effects models tested associations between neural activation to critical (*vs* neutral) feedback and emotions in interpersonal situations. Youth who exhibited higher activation in the sgACC to parental criticism reported less happiness during daily positive interpersonal situations. No significant neural predictors of negative emotions (e.g. sadness and anger) emerged. These findings provide evidence of real-world correlates of neural reactivity to social threat that may have important clinical implications.

Many important socio-emotional changes occur during adolescence, including increases in sensitivity to social cues and feedback (Somerville, 2013; Blakemore and Mills, 2014). Heightened sensitivity to social feedback may help adolescents learn to navigate complex social environments and contribute to identity development—essential tasks during this developmental period. Changes in the salience of positive and negative social feedback during adolescence may be underpinned by maturational change in the brain’s affective salience network (ASN), a network of cortical [e.g. anterior insula (AI) and medial prefrontal cortex] and subcortical (e.g. amygdala) brain regions involved in socio-emotional processing and emotion regulation (Masten *et al.*, 2009; Silk *et al.*, 2014; Butterfield *et al.*, 2021). However, links between ASN activity and daily socio-emotional functioning are understudied. To gain insight into how brain maturation might contribute to adolescent behavior, and vice versa, we need robust research linking neural activity to real-world behavioral measures. The present study addresses this need by linking neural reactivity to parental criticism, a salient form of social feedback, to affective responses during daily interpersonal situations in adolescents with a history of anxiety disorders who are at high risk for future affective psychopathology.

Within the ASN, the subgenual anterior cingulate cortex (sgACC), AI and amygdala play especially important roles in responding to salient social feedback and guiding adolescent socio-emotional behavior (Masten *et al.*, 2009; Lau *et al.*, 2012; Silk *et al.*, 2014). The sgACC is integral to monitoring, regulating and generating negative emotions (e.g. Mayberg, 2003; Siegle *et al.*, 2012), acting as a ‘bridge’ between limbic regions that generate emotions and cortical regions involved in cognitive control (Scharnowski *et al.*, 2020). Research consistently supports the role of the sgACC in emotional responses to social feedback during adolescence (Masten *et al.*, 2009; Rudolph *et al.*, 2016). The amygdala is involved in attentional orientation to emotionally salient cues (e.g. threats and rewards) and learning (Cardinal *et al.*, 2002; Cole *et al.*, 2013). Last, the AI is central to the integration of cognitive and affective information and implicated in subjective ‘feelings’ (Masten *et al.*, 2009; Singer *et al.*, 2009; van Houtum *et al.*, 2022).

Although increases in neural activity within the ASN are developmentally normative during adolescence (Silk *et al.*, 2012), high activation in these regions to social threat specifically (e.g. parental criticism, peer rejection, exclusion or victimization) may contribute to the development of internalizing psychopathology during adolescence, including anxiety (Guyer *et al.*, 2008), depression (Masten *et al.*, 2011; Silk *et al.*, 2014, 2022; Jankowski *et al.*, 2018) and suicidality (Oppenheimer *et al.*, 2020). More research is needed to understand why heightened ASN activation to social threat places youth at risk for internalizing psychopathology. Studying how activity in these regions is related to proximal affective states or real-world functioning would provide not only needed behavioral correlates of neuroimaging measures but also a meaningful context for potentially understanding associations between adolescent brain activity and psychopathology. To maximize the clinical relevance of this work, we examined such brain–behavior correlates in a sample of youth at high risk for psychopathology. More specifically, this sample includes youth with a current and/or past diagnosis of one or more anxiety disorders who received psychotherapy (either cognitive behavioral therapy or supportive psychotherapy) as part of a randomized clinical trial about 3 years prior to presenting data collection (Silk *et al.*, 2018, 2022). Close to one-third of the youth included in the present study maintained an anxiety disorder diagnosis. As youth with a history of anxiety are more neurobiologically sensitive to threats (Britton *et al.*, 2011) and are at increased risk for future psychopathology, particularly depression (Pine *et al.*, 1998; Silk *et al.*, 2022), our investigation into associations between neural sensitivity to social threat and real-world interpersonal experiences may provide important context for understanding why youth with anxiety are at higher risk for future depression.

Much of the research on ASN activation to social threat in adolescence has focused on social threat from peers specifically; however, peers are not the only source of salient social feedback adolescents receive. In addition to feedback from teachers, coaches and mentors, feedback from parents remains important during adolescence and parenting behaviors continue to influence adolescents’ neural, emotional and behavioral functioning (Tan *et al.*, 2014; Oppenheimer *et al.*, 2016; Butterfield *et al.*, 2021). Parental criticism may be a particularly salient form of negative parental feedback—especially during adolescence as youth seek greater independence (Steinberg and Morris, 2001; Steinberg and Silk, 2002). Although parental criticism can be a constructive and important social cue shaping development and future behaviors (Harris and Howard, 1984; Smetana, 1989), it can also represent a threat to autonomy and activate aversive feelings of being diminished or disrespected (Yeager *et al.*, 2018). Parental criticism may have detrimental effects on emotions and self-image and has been linked to psychopathology and self-injurious thoughts and behaviors (e.g. Hooley and Gotlib, 2000; Wedig and Nock, 2007; Burkhouse *et al.*, 2012).

Although typically developing adolescents are neurobiologically sensitive to parental criticism (Lee *et al.*, 2015; van Houtum *et al.*, 2022), heightened activity in the ASN to parental criticism has been linked to internalizing psychopathology during adolescence and adulthood (Hooley *et al.*, 2005, 2009; Aupperle *et al.*, 2016; Silk *et al.*, 2017). Parental criticism may influence youth’s neurocognitive responses to positive and negative environmental cues (e.g. emotional faces and reward/loss; James *et al.*, 2018, 2021). Moreover, high neural responsivity to parental criticism may reflect a history of perceiving greater criticism and/or less warmth from parents (Butterfield *et al.*, 2021), which, as previously described, has been associated with psychopathology. Given the important role of parental criticism in socialization and socio-emotional development, high neural reactivity to parental criticism may lead adolescents to expect or perceive more negative feedback not only from parents (c.f., Hooley *et al.*, 2012; but see also van Houtum *et al.*, 2022), but also from social agents more broadly. Furthermore, high activity in brain regions associated with emotion modulation and regulation, including the amygdala, sgACC and AI, to parental criticism may be associated with adolescents’ emotional and behavioral responding in daily interpersonal situations, especially those with the potential to be negative and for youth who are more reactive to potential threat, including youth with anxiety (Creswell *et al.*, 2005; Bar-Haim *et al.*, 2007). This hypothesis aligns with prior research showing that adolescents with aberrant neural activity to peer rejection report more negative emotional responses during negative interactions with peers (Sequeira *et al.*, 2021b) and have trouble in disengaging attention from critical social feedback (Sequeira *et al.*, 2021a).

In addition to investigating how high ASN activity is associated with affective responses in negative social interactions, we were also interested in how ASN activation to parental criticism impacts youth’s affective responding in positive daily social interactions. Silk *et al.* (2012) propose that hypersensitivity to social threat in youth with a history of anxiety may interfere with the development of reward processing; hypersensitivity to social threat may lead youth to avoid positive situations due to the possibility of threat, thus reducing positive social experiences. Accordingly, in the same sample of youth recruited for the present study, Silk *et al.* (2022) recently showed that higher sgACC activation to peer rejection *vs* acceptance feedback was associated with feeling less close with/connected to peers in daily life. Moreover, higher maternal negative affect is associated with adolescents’ diminished neural reward sensitivity (Tan *et al.*, 2014) and lower self-reported positive affect in daily life (Griffith *et al.*, 2018). Youth with high neurobiological sensitivity to social threat may also be less likely to engage with (and more likely to avoid) potentially positive social interactions (e.g. a school dance) due to the possibility of being rejected (e.g. not being asked to dance). Such avoidance could be physical in nature (i.e. not attending such events) or emotional (i.e. blunting the experience of pleasure/anhedonia), aligning with a contrast avoidance hypothesis (Newman and Llera, 2011). Both types of avoidance are commonly seen in youth with anxiety.

The present study thus examines how neural reactivity to parental criticism is associated with real-world measures of adolescent socio-emotional functioning in daily positive and negative interpersonal situations among adolescents with a history of anxiety disorders. To provide the most ecologically valid and reliable measure of real-world emotions during interpersonal events, we used ecological momentary assessment (EMA), which employs signaling devices to enable the collection of real-time data on emotion and behavior in the nature environment (Larson and Csikszentmihalyi, 1983; Hormuth, 1986; Stone *et al.*, 1999). We predicted that adolescents with a history of anxiety who exhibited greater neural activity to parental criticism (*vs* neutral comments) in the sgACC, amygdala and AI would show altered emotional responses during both positive and negative interpersonal experiences assessed via EMA. We hypothesized that greater neural reactivity within the ASN to parental criticism (*vs* neutral feedback) would be associated with (i) less happiness during day-to-day positive interpersonal situations and (ii) more sadness and anger during day-to-day negative interpersonal situations. In addition to testing these hypotheses, this study contributes more broadly to an under-studied research area linking brain and daily behavior. Multimodal studies bridging laboratory-based brain findings and behavioral responses in everyday life are necessary to improve the generalizability of both methodologies and support the relevance of neuroimaging methodologies in developmental and clinical research. This research is essential to deepening our understanding of socioaffective processes in adolescence.

## Method

### Participants

Participants were 44 youth (females) aged 11–16 years (*M*_age_ = 13.31, standard deviation (SD) = 1.33). The sample was predominately (95.5%) White. Average annual family income was between $60 000 and 80 000 and ranged from $10 000 to $100 000+. Participants had a history of an anxiety disorder and had been previously treated with psychotherapy as part of a randomized clinical trial (Wave 1; Silk *et al.*, 2018). Participants were subsequently enrolled in a follow-up study to examine risk for depression among youth with anxiety; data reported in the current study were collected 2 years following treatment (Wave 2; Silk *et al.*, 2019). Participants in the present study are a subset of participants included in previously published work (Silk *et al.*, 2019, 2022). At the time of Wave 2 data collection, 13 participants included in this present study met criteria for a current anxiety disorder (Table 1). Other current diagnoses included depressive disorder not otherwise specified (*n *= 1), Tourette’s disorder (*n *= 1) and attention deficit/hyperactivity disorder (*n *= 2).

**Table 1.** Demographic and clinical characteristics

	*N*	%	Mean	SD	Range
Sex—female	25	57			
Race					
White	42	95.5			
Black or African American	1	2.3			
Biracial	1	2.3			
Age			13.31	1.33	11.44–16.39
Anxiety symptoms			17.82	11.12	1–43
Depression symptoms			9.09	8.78	0–34
Current anxiety diagnoses					
GAD only	5	11.4			
SocAD only	1	2.3			
SP only	2	4.5			
SEP and SP	1	2.3			
GAD and SocAD	1	2.3			
SocAD and SP	1	2.3			
GAD, SocAD, SP	2	4.5			

*Note:* Demographic and clinical data were assessed at the time point of EMA and fMRI data collection.

GAD = generalized anxiety disorder, SocAD = social anxiety disorder, SEP = separation anxiety disorder, SP = specific phobia.

### Procedure

At Wave 1, youth were randomized to 16 sessions of treatment (cognitive behavioral therapy or a supportive comparison therapy); most exhibited a positive treatment response (Silk *et al.*, 2018 for full procedures for the Wave 1 randomized controlled trial; Clinical Trials No. NCT00774051). Of 133 participants in Wave 1, 105 were enrolled in the follow-up study and returned to the laboratory for Wave 2. The 28 participants who did not enroll in the follow-up study had either dropped out of the original study (*n* = 12) or chose not to participate in the follow-up (*n* = 16). As a part of the follow-up study, a total of 47 participants completed neuroimaging with usable data from the Expressed Emotion task. Fifty participants did not complete the scan because they had gotten dental braces (*n* = 20), refusal (*n* = 17), loss to follow-up (*n* = 1), claustrophobia (*n* = 2) and other reasons that were not documented (*n* = 10). Three participants with usable neuroimaging data failed to complete EMA, resulting in a final sample of 44 youth. A parent or legal guardian provided informed consent, and adolescents provided assent (Human Research Protection Office #PRO07110273).

Diagnoses were determined at all time points by independent evaluators who were blind to original treatment assignment using the Schedule for Affective Disorders and Schizophrenia for School-Age Children-Present and Lifetime Version (Kaufman *et al.*, 1997). Depression symptoms were assessed at all time points using youth report on the long version of the Mood and Feelings Questionnaire-Child report (MFQ-C; Angold *et al.*, 1995). Anxiety symptoms were assessed using youth report on the Screen for Child Anxiety Related Emotional Disorders-Child report (SCARED-C: Birmaher *et al.*, 1997).

### Expressed emotion paradigm

During the functional magnetic resonance imaging (fMRI) scan, participants listened to their parents’ (95.5% mothers) comments about them, delivered via MRI compatible headphones. There were two audio clips for critical, praising and neutral comments, each lasting for 30 s. Similar procedures used in previous studies (Hooley *et al.*, 2005, 2009) were used for obtaining audio clips. Parents generated two 30 s audio clips describing things that bothered them about their child [critical statements beginning with ‘Name, one thing that bothers me about you is…’, i.e. not doing chores or attitudes toward family member(s)], two 30 s audio clips describing things that they especially liked about their child (praising statements beginning with ‘Name, one thing I really like about you is…’,) and two 30 s neutral clips (neutral statements: something your child will not find interesting, e.g. grocery shopping and weather). Parents formulated their critical remarks based on something they had shared with their child on more than one occasion, so youth would not be exposed to new and potentially disturbing information while in the scanner.

As described in Sequeira *et al.* (2019), there was one block each for critical, praising and neutral conditions. Each block (run) consisted of two 30.06 s comment presentations (30 s audio clip with a 0.06 s additional duration to match repetition time (TR) 1.67 s) and three 30.06 s rest periods. Each began with a rest period, followed by one comment presentation, the second rest period, the second (same type of) comment presentation and then the last rest period. To minimize possible emotional carryover after listening to criticism or praise from parents, the neutral block was presented first and the order of two other blocks was counterbalanced across participants. After listening to each clip, participants were asked to rate the emotional intensity of the comment (i.e. how negative the comment was) using a 1–10 scale. The present study focuses only on youth’s responses to their parents’ critical statements relative to neutral comments.

### MRI data acquisition, preprocessing and analysis

Images were acquired using a 3T Siemens Trio scanner. Blood-oxygen-level-dependent functional images were acquired using a T2*-weighted reverse echo planar imaging sequence. Thirty-two 3.2 mm axial slices were acquired parallel to the anterior–posterior commissure line (TR/echo time (TE) = 1670/29 ms, field-of-view (FOV) = 205 mm, flip angle = 75º). Before the start of the fMRI task, a high-resolution T1-weighted magnetization-prepared rapid acquisition gradient echo (MPRAGE) image (1 mm, axial) was collected for each participant.

Images were preprocessed and analyzed using Statistical Parametric Mapping (SPM) version 12. Volumes were manually reoriented to the anterior–posterior commissure line and corrected for slice timing. Images were realigned to correct for motion, segmentation and co-registration to the mean functional image; realigned images were then spatially normalized to standard Montreal Neurological Institute (MNI) template and smoothed with a 6 mm full-width at half-maximum Gaussian filter. Voxels were resampled to be 2 mm^3^. Volumes with motion greater than 5 mm/5º and global intensities more than three standard deviations from the mean were detected using SPM ART toolbox. Data were excluded from analyses if >25% of volumes per session were detected as outliers; based on this cut-off, no participants were excluded. Despiking was completed with interpolation using the ArtRepair toolbox in SPM. Motion parameters were included as regressors in the general linear model design in first-level analyses to correct for slow-drift motion.

First-level analyses included repaired pre-processed volumes, six motion parameters and all conditions from each run (i.e. criticism, praise, neutral, and rest). The contrast included for the current analyses was criticism > neutral. Final analyses used a region-of-interest (ROI) approach. Three ROIs (bilateral amygdala, bilateral AI and bilateral sgACC) were chosen a priori given prior research linking individual differences in activity in these regions in response to social evaluative threat to depression symptoms (Masten *et al.*, 2011; Silk *et al.*, 2014, 2022). The amygdala and AI masks were defined anatomically using the Wake Forest University PickAtlas toolbox; the sgACC mask was defined by multiplying masks for Brodmann areas 34, 24 and 25 by the Neurosynth (http://neurosynth.org) activation map for the term ‘subgenual’. More details on these masks can be found in the Supplementary Data and Silk *et al.* (2022). Parameter estimates for the criticism > neutral contrast were extracted for each ROI (average activation across the ROI) using the MarsBar toolbox for SPM12 and used in correlational and regression analyses in Statistical Package for the Social Sciences (SPSS) version 26, as well as mixed-effects models in R (version 4.0.2).

### EMA

Participants completed an EMA protocol to obtain real-time information on youth’s socio-emotional functioning in the natural environment. Participants were provided answer-only cell phones on which they received calls from research assistants 28 times between 4 p.m. Thursday and 10 p.m. Monday for two consecutive weekends (10 total days). Participants were called at random times within predetermined blocks two times per day after school on Thursdays, Fridays and Mondays and four times per day on Saturdays and Sundays. This schedule was designed to maximize weekend assessments; participants were not contacted during the school day. To assess participants’ emotions during negative experiences at each call, participants were asked, ‘try to remember your thoughts and feelings over the past hour. Think about the time when you felt the worst or the most negative. What happened?’ After identifying an experience, participants were asked whom they were with when the negative emotion occurred (e.g. alone, family member and peer/friend) and to rate how angry and sad they felt at the worst point using a Likert-type scale from 1 (not at all) to 5 (extremely). Participants were also asked to, ‘think about the most enjoyable or happy time in the past hour’, describe whom they were with when the positive emotion occurred and how happy they felt at the best point, using the same Likert-type scale. We focused exclusively on emotional events in which the participant reported the presence of another person (hereafter referred to as ‘interpersonal situations’).

### Analytic plan

Given the repeated measures nature of the outcome variable (positive and negative emotions in interpersonal situations), we ran linear mixed-effects models estimated using restricted maximum likelihood (REML) [‘lme4’ package (Bates *et al.*, 2015)] using R. These models tested associations between neural responses to critical *vs* neutral feedback (fixed effect) and affect in interpersonal situations controlling for time (call number; 1–28). To conserve power with a small sample, no other covariates were added to the main models. However, supplementary sensitivity analyses controlled for anxiety symptoms, depression symptoms, age and gender. Estimation occurred via REML. A random effect of participant was included in all models. Separate models were run for each ROI (sgACC, AI and amygdala) and emotion (i.e. happiness in positive interpersonal situations, anger in negative interpersonal situations and sadness in negative interpersonal situations). Supplemental whole-brain regressions were also conducted in SPM 12 using average emotion ratings as the predictor variables. Clusters surviving a voxel-wise and cluster-wise threshold of *P* < 0.001 are presented later.

## Results

We first examined correlations between emotional intensity of the audio recordings (i.e. criticisms and neutral statements) and youth’s neural reactivity using SPSS, to test whether higher neural activity to criticism with the ASN could be explained by higher negative emotional intensity of the critical or neutral statements. Importantly, there were no significant correlations between the participant-rated negative emotional intensity of the audio recordings and activity in the sgACC, AI and amygdala (all *P* > 0.10).

Associations between youth’s neural activity to parental criticism and day-to-day emotions during positive and negative interpersonal situations were examined using the linear mixed-effects models described earlier. The final models included 708 observations from 44 participants. The only significant predictor of happiness in positive interpersonal situations was sgACC activation to critical (*vs* neutral) feedback (*B* = −0.11, standard error (SE) = 0.05, *P *= 0.045), such that higher sgACC activation to critical (*vs* neutral) feedback was associated with less happiness in positive interpersonal situations. Model results are shown in Table 2. In supplementary sensitivity analyses, the effect of sgACC activity on happiness in positive interactions was consistent when controlling for anxiety and depression symptoms (*B* = −0.11 and *P *= 0.0496) and when controlling for age and gender (*B* = −0.11 and *P *= 0.058). No covariates (anxiety, depression, age and gender) were independently associated with daily happiness in positive interpersonal situations (*P* > 0.46) or sgACC activity (*P* > 0.20). Additional sensitivity analyses are presented in the supplement.

**Table 2.** Summary of results for neural activation predicting happiness in positive interpersonal situations

	Estimate	SE	d*f*	*t*	*P*
sgACC					
Fixed effects					
Intercept	3.90	0.10	66.9	40.9	<0.001
Time	0.00	0.00	666.7	0.07	0.947
sgACC activity	−0.11	0.05	42.1	−2.06	0.045
	Variance	SD			
Random effects					
ID (intercept)	0.29	0.53			
Residual	0.41	0.64			
Amygdala					
Fixed effects					
Intercept	3.90	0.10	66.2	40.0	<0.001
Time	0.00	0.00	667.1	0.09	0.933
Amygdala activity	−0.07	0.05	42.4	−1.43	0.159
	Variance	SD			
Random effects					
ID (intercept)	0.30	0.55			
Residual	0.41	0.64			
AI					
Fixed effects					
Intercept	3.90	0.10	64.4	39.1	<0.001
Time	0.00	0.00	666.8	0.08	0.936
AI activity	−0.02	0.06	42.0	−0.39	0.697
	Variance	SD			
Random effects					
ID (intercept)	0.32	0.56			
Residual	0.41	0.64			

Neither amygdala activation to critical (*vs* neutral) feedback (*B* = −0.07, SE = 0.05 and *P *= 0.159) nor AI activation to critical (*vs* neutral) feedback (*B* = −0.02, SE = 0.06 and *P *= 0.697) were significantly associated with happiness in positive interpersonal situations (Table 2). No significant neural predictors of negative emotions (i.e. sadness and anger) in response to negative interpersonal situations emerged (*P* > 0.05; Tables S1 and S2, Supplementary Data).

**Fig. 1.**
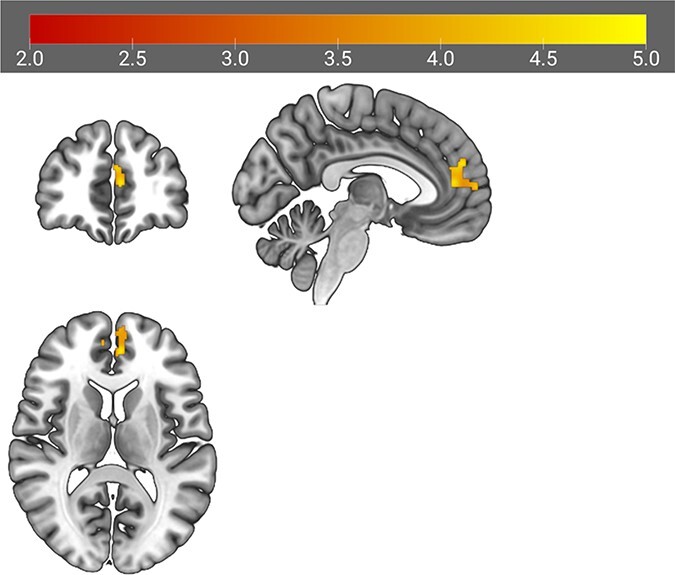
PFC cluster resulting from supplemental whole-brain analyses. *Note:* Color bar depicts *t*-values. Results thresholded at *P* < 0.001 (voxel and cluster level).

In supplemental whole-brain analyses, one significant negative association emerged between average daily happiness in positive interpersonal situations and activation in a region of the left prefrontal cortex, which included portions of the left dorsal ACC (dACC), dorsolateral prefrontal cortex (dlPFC) and anterior PFC [cluster size = 3352 mm^3^; peak activation (MNI *x*, *y*, *z*) at −6, 46, 12; −6, 52, 26; and −6, 60, 8; Zs = 3.98–4.38; *P* < 0.001; Figure 1). Greater activation in this prefrontal region to critical (*vs* neutral) feedback was associated with lower average happiness in positive interpersonal situations in daily life.

### Post hoc analyses

To test specificity of the brain–behavior relationship, sgACC activation to parental criticism (*vs* neutral statements) was run as a predictor of happiness in daily interpersonal situations involving moms and peers separately. SgACC activity did not significantly predict happiness in situations with mom (*B* = −0.08, SE = 0.06 and *P* = 0.174 with 318 observations from 43 participants) but did significantly predict happiness in peer situations (*B* = −0.15, SE = 0.07 and *P* = 0.037 with 173 observations from 36 participants). Note that not all participants were included in analyses as some did not report experiences with mom or peers. Additionally, to test specificity to neural responses to parental criticism (*vs* salient child-focused statements more generally), sgACC activation to parental praise (*vs* neutral statements) was also run as a predictor of happiness in daily interpersonal situations; sgACC activation to praise was not a significant predictor of happiness (*B* = −0.10, SE = 0.07, *t* = −1.51 and *P* = 0.138).

As discussed in the introduction, we hypothesized that in youth with a history of anxiety, high sgACC activation to criticism may be associated with lower happiness in daily positive social interactions due to greater avoidance of these situations and/or greater trait anhedonia. In post hoc exploratory analyses, correlations between sgACC activation to criticism, happiness in daily positive interactions and a trait measure of anhedonia in youth (the Pleasure Scale for Children; Kazdin, 1989) were run. A small but non-significant correlation between sgACC activation to parental criticism and anhedonia was found (*r* = −0.22 and *P* = 0.160), and average happiness in daily positive interactions was strongly associated with the trait measure of anhedonia (*r* = 0.62 and *P* < 0.001). The percentage of positive and negative situations spent with others (*vs* alone) was used as a proxy for social engagement, with low social engagement potentially (though not definitively) indicative of higher social avoidance. Social engagement was not significantly associated with sgACC activation to parental criticism (*r* = −0.04 and *P* = 0.788) or average happiness in daily positive social interactions (*r* = 0.20 and *P* = 0.202).

## Discussion

Supporting our hypothesis, adolescents who exhibited greater reactivity to parental criticism in the sgACC reported less happiness during day-to-day positive interpersonal situations. This link between sgACC activation to parental criticism and daily emotional experiences during interpersonal situations only emerged for happiness during positive interpersonal situations, particularly positive situations with peers, which could index anhedonic symptoms of depression in these youth. Neither amygdala nor AI activation to parental criticism were associated with happiness in positive social situations.

Current findings bolster research showing neural activation to social evaluative threat plays a key role in risk for internalizing symptoms. Several studies have demonstrated group differences in neural responses within the ASN to parental criticism among those with and without depression symptoms (Hooley *et al.*, 2005, 2009; Aupperle *et al.*, 2016; Silk *et al.*, 2017). The sgACC consistently emerges as a key neural region activated in response to peer rejection, exclusion and victimization (Masten *et al.*, 2011; Silk *et al.*, 2014, 2022; Rudolph *et al.*, 2016; Jankowski *et al.*, 2018). As previously described, the sgACC is integral to monitoring, regulating and generating negative emotions (e.g. distress and sadness; Mayberg, 2003; Siegle *et al.*, 2012) and involved in emotional responses following social feedback (Somerville *et al.*, 2006; Masten *et al.*, 2009; Sebastian *et al.*, 2011; Rudolph *et al.*, 2016). The sgACC is a key region in the neural circuitry supporting emotion regulation given its dense structural connections to the amygdala and prefrontal regions that play a role in top-down regulation (e.g. the dorsomedial prefrontal cortex) [see Scharnowski *et al.* (2020) for a review]. Moreover, the prefrontal cortex may exert its regulatory effects on the amygdala through the sgACC (Scharnowski *et al.*, 2020). Higher sgACC activation to parental criticism may thus be indexing over-regulation of a subcortical response to criticism, which could support blunted affective responses in daily life. This may be especially relevant during adolescence, considering the maturation in emotion regulation neural circuitry occurring during this time.

Although present findings should be interpreted with some caution due to the small sample size, supplemental whole-brain analyses linking happiness in positive social interactions to dACC activation to parental criticism may bolster these findings. Like the sgACC, the dACC and surrounding dlPFC have dense structural connections to other brain regions involved in emotion regulation, including the insula, ventral striatum and amygdala (Heilbronner and Hayden, 2016), and may downregulate the amygdala (Ochsner *et al.*, 2012). The dACC also commonly activates to social evaluative threat (Rotge *et al.*, 2015). Like the sgACC finding, higher dACC activation may also represent over-regulation of subcortical responses to criticism; in the real world, youth with this pattern of brain activity may be more likely to over-regulate their emotional responses in salient situations, even positive situations.

Higher sgACC activation to social threat has been linked repeatedly to higher depression symptoms (e.g. Gotlib *et al.*, 2005; Masten *et al.*, 2009; Silk *et al.*, 2014, 2022), and in the current study, a small but nonsignificant correlation emerged between sgACC activity and a trait measure of anhedonia. The current study did not test associations between sgACC activity, daily emotions in interpersonal situations and depressive symptoms due to the small sample size. However, when considered with findings from Silk *et al.* (2022), which employed the same sample, present findings could suggest that the sgACC confers risk for depression by modulating positive emotional reactivity in daily life. Of course, this remains speculative. Notably, present findings could be specific to youth with a history of anxiety disorders, given the nature of the present sample. Nonetheless, it warrants mention that youth with a history of anxiety disorders are at higher risk for the development of depression (Cummings *et al.*, 2014), further underscoring the importance of conducting research in this population.

No significant associations emerged between youth’s neural responses to parental criticism and their experience of sadness or anger during negative interpersonal situations. Disruptions in socio-emotional functioning among youth with heightened sgACC reactivity to parental criticism may be specific to deficits in positive emotional responding during positive interpersonal situations rather than to elevations in negative emotional responding during negative interpersonal situations; this interpretation aligns with the previously discussed role of the sgACC in downregulation. When youth expect or perceive higher levels of social threat, they may learn to temper their own emotional engagement during interpersonal situations to reduce the impact of any threat. This avoidance may be especially relevant during positive interpersonal experiences. Indeed, in positive contexts, youth with anxiety disorders may guard themselves (e.g. by prolonging negative emotions or dampening positive emotions) against unexpected negative events (e.g. social threat) that cause jarring, negative emotional shifts, whereas, in negative contexts, social threat (and resultant negative emotions) is already anticipated and experienced (Newman and Llera, 2011). Although strategies like avoidance or disengagement could be adaptive in terms of short-term emotion regulation or in the context of negative interpersonal interactions, these strategies are maladaptive in the context of positive social interactions and could have more enduring consequences. Present findings seem to align with prior research, showing that objective and perceived measures of parental negative affect, constructs related to criticism, have a stronger impact on adolescents’ positive affect and neural reward circuitry than negative affect and threat circuitry (Tan *et al.*, 2014; Griffith *et al.*, 2018).

By integrating fMRI and EMA, the current study contributes to the nascent body of research identifying real-world correlates of neuroimaging findings. As one of the only studies to link neural responses to parental criticism and EMA, the current work extends previous efforts to understand social threat–related brain–behavior associations during adolescence, which have focused largely on peer rejection and exclusion (Masten *et al.*, 2012; Sequeira *et al.*, 2021b; Silk *et al.*, 2022). Silk *et al.* (2022) found that increased sgACC reactivity to social threat was highly correlated with lower feelings of peer closeness, which converges with the primary finding of the current study. To date, only one other study has examined the relation between neural responses to parental criticism and day-to-day behavioral responses (van Houtum *et al.*, 2022). Although van Houtum and colleagues showed that greater perceived daily parental warmth was linked to more positive mood during the parental feedback task, no significant relations between perceived daily parental criticism and mood during the task emerged. Moreover, there were no significant associations between neural responses during the feedback task and perceived parental warmth or criticism in daily life (van Houtum *et al.*, 2022). These investigations are essential to identifying potential mechanisms underlying risk and specific targets for intervention during sensitive developmental windows.

Strengths of the current study include the use of an ecologically valid fMRI task and EMA to capture day-to-day experiences of emotions during interpersonal situations. Nonetheless, several limitations warrant mention. First, our sample size likely precluded the detection of small to medium effects. It is possible the effects seen here are inflated (Marek *et al.*, 2022) and should thus be interpreted with caution; however, our study is bolstered by the collection of high-quality behavioral data using EMA. Moreover, it is notable that associations between depression symptoms and activation in the sgACC to social threat have now been replicated across several studies (Gotlib *et al.*, 2005; Masten *et al.*, 2009; Silk *et al.*, 2014, 2022). The nature of our sample (youth previously treated for an anxiety disorder) was a unique strength, as this population is at increased risk for future psychopathology (Silk *et al.*, 2022); however, when interpreting the present findings, it is important to note that most youth did not meet diagnostic criteria for a current anxiety disorder or report clinically significant levels of anxiety or depression symptoms.

It is important to note that we did not measure the frequency or chronicity of actual parental criticism in youth’s daily lives. Although our study provides new information about how youth who exhibit elevated sgACC activation in response to parental criticism experience happiness during daily social situations, it remains unclear how the presence of a critical parent or exposure to chronic parental criticism impacts youth’s neural responses to parental criticism during this task. Research is needed to determine whether youth who experience routine parental criticism show similar patterns of emotional responding during daily interpersonal situations as the youth who exhibit greater sgACC activation to parental criticism in the current study. Similarly, it is possible that other parental characteristics, including psychopathology, play a role in our findings. Although the ecological validity of our fMRI task was a strength, this task confounds social stimuli and threatening stimuli, and it is impossible to know whether individual differences in sgACC activity are indexing sensitivity to threat more generally or social threat specifically. It would be interesting to replicate the present findings using non-social punishing stimuli (e.g. monetary loss). Furthermore, we did not measure adolescents’ experience of negative emotions during the positive situations or positive emotions during the negative situations. Therefore, we cannot disentangle whether our results were specific to the emotion (i.e. happiness), context (i.e. positive social situations) or combination of the two. Finally, our small sample was predominately White, limiting generalizability.

Overall, these findings indicate that youth’s cortical reactivity to parental criticism, a salient social evaluative threat, is linked to important differences in day-to-day experiences of emotion during positive interpersonal situations. Specifically, youth who exhibit greater activation in the subgenual and dorsal ACC to parental criticism report less happiness in positive interpersonal situations in their daily lives. Multimodal investigation linking laboratory-based neuroimaging findings with tangible, everyday behavior in naturalistic settings has important implications for the generalizability and clinical relevance of studies that employ fMRI and EMA methodologies. Moreover, our results provide preliminary support for a valuable, real-world correlate of fMRI findings that, if replicated in future longitudinal and mechanistic research, could provide specific targets for a new generation of interventions.

## Supplementary Material

nsad020_Supp

## Data Availability

The data that support the findings of this study are available from the corresponding author upon reasonable request.
