# Supplementary material for: Adolescents’ neural reactivity to parental criticism is associated with diminished happiness during daily interpersonal situations
Source: Soc Cogn Affect Neurosci. 2023 Apr 1;18(1):nsad020. doi: 10.1093/scan/nsad020 (PMC10099162; doi:10.1093/scan/nsad020)
Supplement: nsad020_Supp [file nsad020_supp.zip › scan-22-118-File003.docx]

**Neural reactivity to parental criticism is associated with diminished happiness**

**during daily interpersonal situations**

***Supplemental Information***

**Creation of ROI Masks**

The bilateral amygdala and bilateral AI anatomical ROIs were created using the AAL, Talairach Daemon, and IBASPM 71 atlases in the WFU PickAtlas toolbox for SPM12. The sgACC ROI was created using Neurosynth (http://neurosynth.org) and FSL v6.0.3. One anatomical mask of regions encompassing the bilateral subgenual, medial, and/or inferior portions of Brodmann areas 34, 24, and 25 was created by downloading separate masks for each region defined by the Talairach Daemon Labels in FSLeyes (v0.31.2) and adding these masks together using the fslmaths function. This mask was then multiplied by the Neurosynth activation map for the term “subgenual” to ensure the specificity of the ROI. The activation map was thresholded at FDR-corrected *p*<.001 by default.

**Sensitivity Analyses**

Restricting analyses to the 35 participants whose EMA collection occurred within 60 days prior to the fMRI scan, findings were consistent though non-significant, likely related to the reduction in power (sgACC B=-.12, SE=.06, *t*=-1.96, *p*=.059).

**Table 1S.** *Summary of Results for Neural Activation Predicting Sadness in Negative Interpersonal Situations.*

| ***Subgenual Anterior Cingulate Cortex*** | | | | | |
| --- | --- | --- | --- | --- | --- |
|  | **Estimate** | **SE** | ***df*** | ***t*** | ***p*** |
| **Fixed Effects** |  |  |  |  |  |
| Intercept | 2.08 | .15 | 67.8 | 13.67 | <.001 |
| Time | .01 | .01 | 436.5 | 1.53 | .126 |
| sgACC Activity | -.05 | .08 | 39.7 | -.55 | .585 |
|  | **Variance** | **SD** |  |  |  |
| **Random Effects** |  |  |  |  |  |
| ID (Intercept) | .65 | .81 |  |  |  |
| Residual | .88 | .94 |  |  |  |
|  |  |  |  |  |  |
| ***Amygdala*** | | | | | |
|  | **Estimate** | **SE** | ***df*** | ***t*** | ***p*** |
| **Fixed Effects** |  |  |  |  |  |
| Intercept | 2.08 | .15 | 68.1 | 13.78 | <.001 |
| Time | .01 | .01 | 436.6 | 1.54 | .124 |
| Amygdala Activity | -.08 | .08 | 36.6 | -1.08 | .286 |
|  | **Variance** | **SD** |  |  |  |
| **Random Effects** |  |  |  |  |  |
| ID (Intercept) | .64 | .80 |  |  |  |
| Residual | .88 | .94 |  |  |  |
|  |  |  |  |  |  |
| ***Anterior Insula*** | | | | | |
|  | **Estimate** | **SE** | ***df*** | ***t*** | ***p*** |
| **Fixed Effects** |  |  |  |  |  |
| Intercept | 2.10 | .15 | 68.1 | 13.97 | <.001 |
| Time | .01 | .01 | 436.2 | 1.49 | .137 |
| AI Activity | -.12 | .09 | 39.5 | -1.38 | .177 |
|  | **Variance** | **SD** |  |  |  |
| **Random Effects** |  |  |  |  |  |
| ID (Intercept) | .62 | .79 |  |  |  |
| Residual | .88 | .94 |  |  |  |

**Table 2S.** *Summary of Results for Neural Activation Predicting Anger in Negative Interpersonal Situations.*

| ***Subgenual Anterior Cingulate Cortex*** | | | | | |
| --- | --- | --- | --- | --- | --- |
|  | **Estimate** | **SE** | ***df*** | ***t*** | ***p*** |
| **Fixed Effects** |  |  |  |  |  |
| Intercept | 2.45 | .15 | 57.1 | 15.93 | <.001 |
| Time | .00 | .00 | 431.4 | .29 | .774 |
| sgACC Activity | .01 | .09 | 39.8 | .13 | .899 |
|  | **Variance** | **SD** |  |  |  |
| **Random Effects** |  |  |  |  |  |
| ID (Intercept) | .76 | .87 |  |  |  |
| Residual | .63 | .80 |  |  |  |
|  |  |  |  |  |  |
| ***Amygdala*** | | | | | |
|  | **Estimate** | **SE** | ***df*** | ***t*** | ***p*** |
| **Fixed Effects** |  |  |  |  |  |
| Intercept | 2.45 | .15 | 57.1 | 15.92 | <.001 |
| Time | .00 | .00 | 431.4 | .39 | .774 |
| Amygdala Activity | .01 | .08 | 39.7 | .12 | .904 |
|  | **Variance** | **SD** |  |  |  |
| **Random Effects** |  |  |  |  |  |
| ID (Intercept) | .76 | .87 |  |  |  |
| Residual | .63 | .80 |  |  |  |
|  |  |  |  |  |  |
| ***Anterior Insula*** | | | | | |
|  | **Estimate** | **SE** | ***df*** | ***t*** | ***p*** |
| **Fixed Effects** |  |  |  |  |  |
| Intercept | 2.45 | .15 | 56.7 | 15.8 | <.001 |
| Time | .00 | .00 | 431.0 | .29 | .775 |
| AI Activity | .00 | .09 | 39.7 | -.04 | .970 |
|  | **Variance** | **SD** |  |  |  |
| **Random Effects** |  |  |  |  |  |
| ID (Intercept) | .77 | .88 |  |  |  |
| Residual | .63 | .80 |  |  |  |
